# Management and outcomes in acute symptomatic hernias: a retrospective cohort study and review of cross-over to surgery

**DOI:** 10.1007/s00423-026-04126-y

**Published:** 2026-07-02

**Authors:** Yichen Liu, Hiba Al-khaffaji, Huarui Xue, Wen Yuan Chung, Hassan Nassar, Saman Habib, Molly Castagna, James Wolff

**Affiliations:** 1 Leicester Medical School, University of Leicester, Leicester, UK; 2 Department of Surgery, University Hospitals of Leicester NHS Trust, Leicester, UK

**Keywords:** Hernia, Acute hernia management, Watchful waiting, Surgery cross-over, Re-presentation, Elective hernia repair

## Abstract

**Purpose:**

Hernias are a frequent cause of acute surgical admission, yet optimal management remains uncertain. Watchful waiting (WW) and waiting-list delays may increase symptom recurrence and surgical crossover, creating uncertainty about when timely elective repair is indicated. This study evaluates acute hernia presentations and how different management pathways may influence outcomes.

**Methods:**

A retrospective cohort study was conducted on 105 patients presenting with symptomatic hernias to the University Hospitals of Leicester from period of September 2024 to January 2025 with patient follow-up in September 2025. Outcomes analysed included length of hospital stay, complications, surgical crossover rates, and re-presentations.

**Results:**

A total of 105 patients met the inclusion criteria. 51(49%) patients were conservatively managed with WW, and 43(41%) listed for elective repair, and 11(10%) underwent emergency surgery at presentation. WW patients had higher re-presentation rates (35.3 vs. 14%, *p* = 0.0172) and surgical crossover rates (33.3 vs. 2.3%, *p* < 0.001) relative to those awaiting elective surgery. In the WW cohort, crossover occurred in 50% of inguinal hernias (9.1% to emergency; 40.9% to elective) and approximately 20.7% of ventral hernias (10.3% to emergency; 10.3% to elective). Emergency hernia repair was associated with increased length of stay compared to elective repair for both ventral (median 4 vs. 0.5 days, *p* = 0.0079) and inguinal hernias (median 1.5 vs. 0 days, *p* < 0.001).

**Conclusion:**

Observed surgical crossover and re-presentation among patients managed with watchful waiting may indicate the importance of identifying patients who are less suitable for prolonged non-operative management. Structured follow-up and reassessment may improve watchful waiting pathways and support shared decision-making regarding the appropriateness of surgical repair. Further studies are needed to validate these findings.

## Introduction

Hernias are a common cause of acute general surgery admission, either as new diagnoses or recurrence with worsening symptoms and complications. Based on NHS England data, almost 100,000 hernia repairs are performed annually, comprising both groin and abdominal wall/ventral hernia repairs [1].

Over recent decades, the European Hernia Society (EHS) has developed numerous guidelines on the treatment and prevention of hernias to improve and standardise hernia care. Surgery is recommended for symptomatic groin hernias, whereas conservative watchful waiting (WW) is reserved for selected asymptomatic or minimally symptomatic patients [2]. For ventral hernias, repair is advised when defect is enlarging and associated with symptoms, but the evidence for watchful waiting remains limited with relatively weaker supporting studies [3–5]. Emergency repairs are generally advocated for complicated hernias with signs of strangulation due to the risk of non-viable bowels if delayed treatment [6].

However, clinical uncertainty may arise, as patient presentations can be heterogenous and vary in both symptom profile and functional impact [7]. For patients who present acutely to emergency surgical services with hernia-related symptoms, with or without features such as strangulation or bowel obstruction that would require emergency surgery, the optimal management strategy may remain insufficiently explored. Existing randomised evidence largely concerns asymptomatic or minimally symptomatic hernias and surgical techniques, while recent UK-based Management of Acutely Symptomatic Hernia (MASH) study further reports minimal evidence and variation in practice for acutely symptomatic groin and abdominal wall hernias [2, 8]. Moreover, in practice, clinician decisions regarding suitability for surgery are further influenced by numerous factors, including but not limited to co-morbidities, age, frailty, local surgical capacity and defect complexity [9, 10].

The aim of this study is to evaluate outcomes associated with management pathways for patients presenting with acute symptomatic hernias, including surgical crossover, re-presentation, and postoperative results. We further seek to identify patient and presentation-level factors associated with progression to surgery and management selection, and to explore whether outpatient follow-up and earlier surgical intervention may optimise care. To contextualise our findings, we also reviewed the current literature on surgical cross-over rates in hernia management. 

## Methods

### Study design and setting

This retrospective study recruited patients at the University Hospitals of Leicester between 02 September 2024 and 03 January 2025 with follow-up in September 2025. The study received approval from The Institutional Review Board of the University Hospitals of Leicester NHS Trust with the number #13,806. It is also reported in accordance with the STROBE guidelines [11].

### Cohort selection

All adult patients presenting to the Surgical Assessment Unit (SAU) during the study period with suspected acute symptomatic hernia were identified. Acute symptomatic hernia presentation was defined as an unplanned presentation to the emergency surgical assessment unit during the study period for a hernia with either (i) hernia-related symptoms sufficiently severe to prompt emergency assessment, with or without limitation of usual activities, and/or (ii) features of a complicated hernia, classified according to standard practice as irreducibility/incarceration, bowel obstruction, or strangulation. Management classification was based on the first acute presentation within the study period, prior management outside this timeframe was not captured.

### Inclusion and exclusion criteria

All eligible adult patients with acute symptomatic hernias were included in this study. Exclusions included patients with no confirmed hernias, duplicated entries, those with hiatal hernias (due to differing management), individuals who died from non-hernia-related causes, and patients attending solely for elective repair without acute presentation during the study period.

### Data collection

Patient list was provided by the hospital audit department. Data were extracted retrospectively from hospital records to capture demographics, hernia characteristics, management pathway, imaging, and complications. Management strategy was defined as watchful waiting (non-surgical, symptom only management), elective-listed/awaiting elective (added to waiting list for day case repair), and emergency surgery (performed during the same admission). Patients were followed up in September 2025 to ascertain final management status and any re-presentations. Hernia type was classified anatomically into inguinal and ventral hernia groups for further analysis. Ventral hernia included umbilical/para-umbilical, epigastric, incisional, parastomal and spigelian hernia. For patients who subsequently underwent elective and emergency surgery, operative data included procedure type, length of stay, and postoperative complications were collected. Postoperative complications were defined as documented adverse events occurring after operative repair. Complications recorded in this study included wound infection, seroma, intra-abdominal collection, anastomotic leak, large bowel obstruction, ileus, and urinary retention.

### Primary objective

To evaluate outcomes across the three management pathways for acute symptomatic hernias. This includes (i) surgical crossover and re-presentation rate in the WW group and in patients awaiting elective repair, and (ii) comparison of postoperative outcomes between patients who underwent elective versus emergency surgery by the end of the follow-up period.

Surgical crossover is when a patient converts from the original management plan to surgery—either from watchful waiting to elective or emergency repair, or from planned elective repair to emergency surgery before the scheduled operation.

### Secondary objective

To compare baseline demographics and clinical differences across the three management cohorts at presentation, identify factors associated with clinician decision-making, and assess variables that may influence outcomes and risk stratification.

### Statistical analysis

Normality of continuous variables was assessed within each management group using the Shapiro–Wilk test. Categorical variables were analysed using Fisher’s exact test due to small cell counts in certain cohorts. Continuous variables including age, BMI, Charlson-Comorbidity Index, length of stay were summarised as median (interquartile range) and analysed using non-parametric test including Kruskal-Wallis and Mann-Whitney U test. Length of stay was recorded in days, with same-day discharge coded as 0 days. One patient who underwent elective surgery had an unusually prolonged length of stay, a sensitivity analysis excluding this case was also performed to determine whether it materially affected the results.

To account for potential confounding, a multivariable multinomial logistic regression analysis was performed on age, gender, co-morbidities using the Charlson-Comorbidity Index (excluding age), hernia types and previous history of hernias. Odds ratios (OR) and 95% confidence interval (CI) were calculated, along with p-values to evaluate the association between selected predictors and perceived management group allocation method (WW, elective-listed and emergency surgery). Watchful waiting was listed as the reference category, as this represented the conservative management baseline against which elective-listed and emergency pathways could be compared.

Time-to-event outcomes were analysed using Kaplan–Meier analysis, and survival curves were compared using the log-rank test. Statistical analyses were performed using GraphPad Prism version 11.0.0 and IBM SPSS Statistics version 31.0.2.0. A p value < 0.05 was considered statistically significant.

## Results

Flow diagram showing the number of patients assessed for eligibility, exclusion (with reasons), and inclusion in the final analysis and management pathways.

**Fig. 1:**
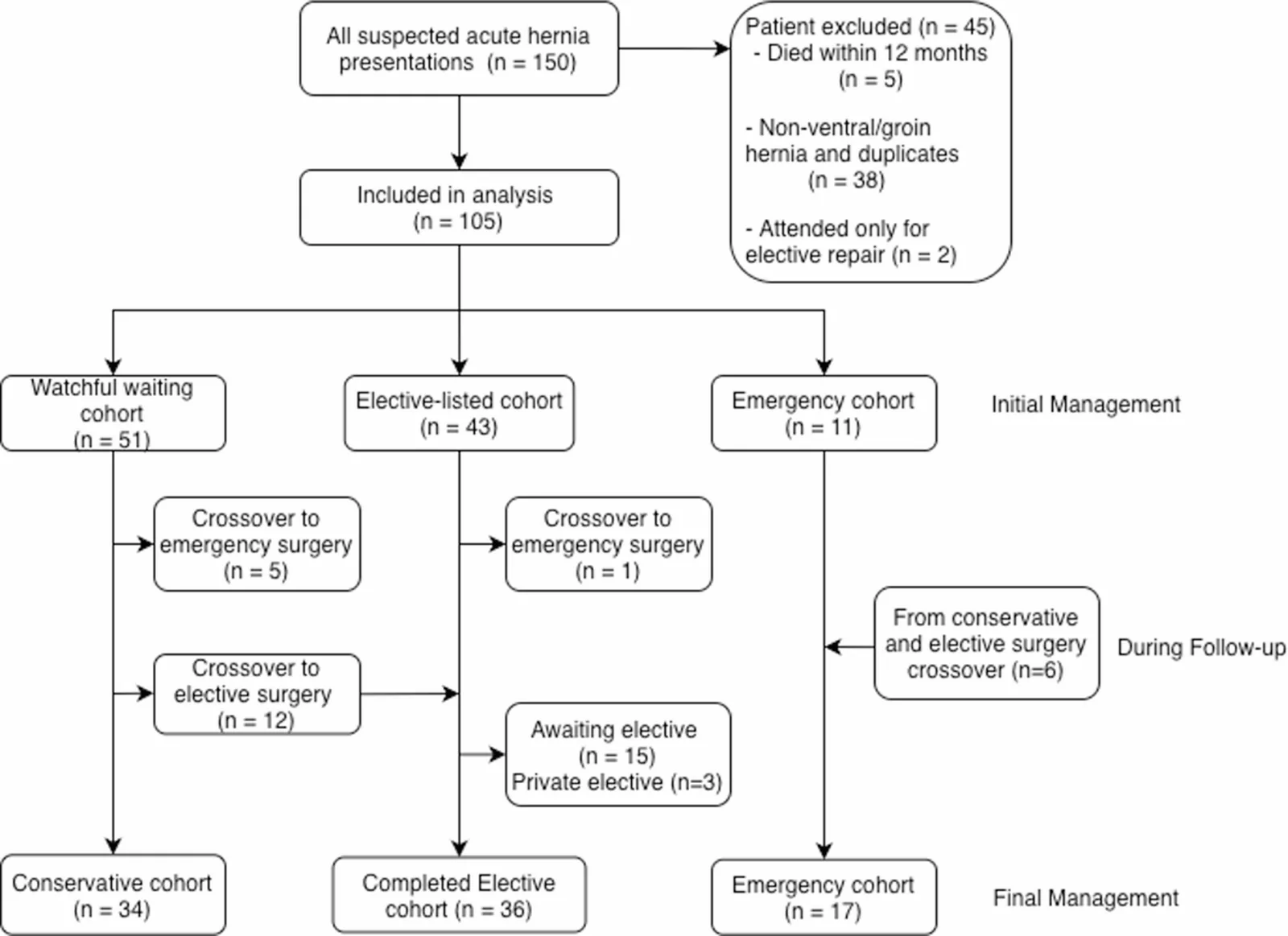
Flow diagram of patient management pathways

A total of 150 patients presented to the surgical assessment unit with suspected hernia during the study period. Following exclusions, 105 patients met the inclusion criteria and were analysed. Of these, 49% (*n* = 51) were managed with watchful waiting, 41% (*n* = 43) were elective-listed, and 10% (*n* = 11) underwent emergency surgery at presentation. Within the WW group, some patients were discharged without planned follow-up, while others were discharged with arrangements for outpatient clinic review, ultrasound, or were provided with open access to the surgical assessment unit. A minority (7.8%, *n* = 4) of WW patients were offered the choice of elective repair at the time but opted for non-surgical option. Patients were followed up and by the end of the study period, 34 patients remained under watchful waiting, 36 underwent elective surgery and 17 underwent emergency surgery, including patients who crossed over from their management pathway (Fig. 1).

Demographic characteristics for all patients included are given in (Table 1). There was a male predominance for all management cohorts (*n* = 80, 76.2%). Baseline age varied significantly between management groups, with the highest median age observed in the watchful waiting group: 64(54–79), 57(42–68) in the elective-listed group and 61(51–82) in the emergency group (*p* = 0.015). More than half of the patients (*n* = 66, 62.9%) had a previously known hernia prior to their recorded presentation to the hospital within the study period.

**Table 1:** Patient demographics upon presentation by management strategy

Age (median IQR)	Watchful Waiting (*n* = 51)	Elective-listed (*n* = 43)	Emergency (*n* = 11)	*p* value*
	64(54–79)	57(42–68)	61(51–82)	0.0150**
BMI (median IQR)	25.89 (23.15–34.15)	28.70 (24.70–34.05)	31.80 (22.80–34.50)	0.5872**
Gender				0.7989
Female	13	9	3	
Male	38	34	8	
Co-morbidities				
Diabetes (T2DM)	11	9	1	0.7804
Hypertension	18	14	5	0.7338
Charlson Co-morbidity Index	3 (1–4)	2 (1–3)	3 (1–5)	0.1432**
Hernia types				
Inguinal	22	20	4	
Umbilical/Para-umbilical	13	20	5	
Incisional	8	2	1	
Para-stomal	5	1	0	
Epigastric	2	0	1	
Spigelian	1	0	0	
Previously known hernia before presentation	34	26	6	0.6766
Emergency features at presentation n%	3(5.9%)	4(9.3%)	11(100%)	**< 0.001**
Strangulation	0	1	7	
Incarceration	1	2	2	
SBO	2	1	2	

Median IQR (interquartile range), *p *value *Fisher’s exact test, except ** Non-parametric Kruskal–Wallis test, SBO = Small Bowel Obstruction

All patients who underwent emergency surgery presented with non-reducible hernias and acute complications. Inguinal hernias comprised 43.8% (*n* = 46) of cases, whereas ventral hernias accounted for 56.2% (*n* = 59). Amongst WW patients and elective-listed patients who presented with acute features (e.g. incarceration, strangulation, or small bowel obstruction), the one patient with a strangulated hernia declined emergency surgery and opted for elective management. Three patients had documented features of SBO but were managed non-operatively following clinical assessment. These included one chronic complex incisional hernia with a broad-neck defect and two reducible hernias: one parastomal and one para-umbilical associated with high BMI. In all three cases, obstructive features were clinically resolving after active non-operative SBO management, with no documented evidence of strangulation or bowel ischaemia. They were therefore closely monitored and followed up according to clinical risk, rather than undergoing immediate emergency repair.

Predictors of management group allocation (WW, elective-listed or emergency surgery) are presented in Table 2. On adjusted analysis, increasing age was the only significant predictor of management group allocation. Patients in the watchful waiting cohort were older than those in the elective-listed cohort. In adjusted analysis, increasing age was associated with lower odds of elective-listed management relative to watchful waiting (aOR 0.957, 95% CI 0.929–0.986; *p* = 0.004). Sex, CCI, hernia type and previously known hernia were not significant predictors of management group. When stratified by hernia types (inguinal and ventral), increasing age remained predictive of WW compared with elective surgery among patients with ventral hernias (aOR 0.937, 95% CI 0.895–0.980; *p* = 0.005). No variables were significantly predictive of watchful waiting among patients with inguinal hernias.

**Table 2:** Multivariable multinomial logistic regression analysis of factors associated with different management groups

Variable	Elective-listed vs. Watchful Waiting aOR (95% CI)	*p* value	Emergency vs. Watchful Waiting aOR (95% CI)	*p* value
Age, per year	0.957 (0.929–0.986)	0.004	0.995 (0.951–1.041)	0.831
Male sex vs. female	0.983 (0.317–3.049)	0.976	0.909 (0.179–4.626)	0.909
Previously known hernia, yes vs. no	0.702 (0.279–1.765)	0.452	0.608 (0.156–2.373)	0.474
Charlson Comorbidity index (excluding age)	1.057 (0.700-1.597)	0.791	1.018 (0.561–1.847)	0.954
Hernia type (Inguinal vs. Ventral)	2.074 (0.743–5.788)	0.163	0.962 (0.199–4.641)	0.962

*aOR *Adjusted odds ratio, *CI *Confidence interval

**Fig. 2:**
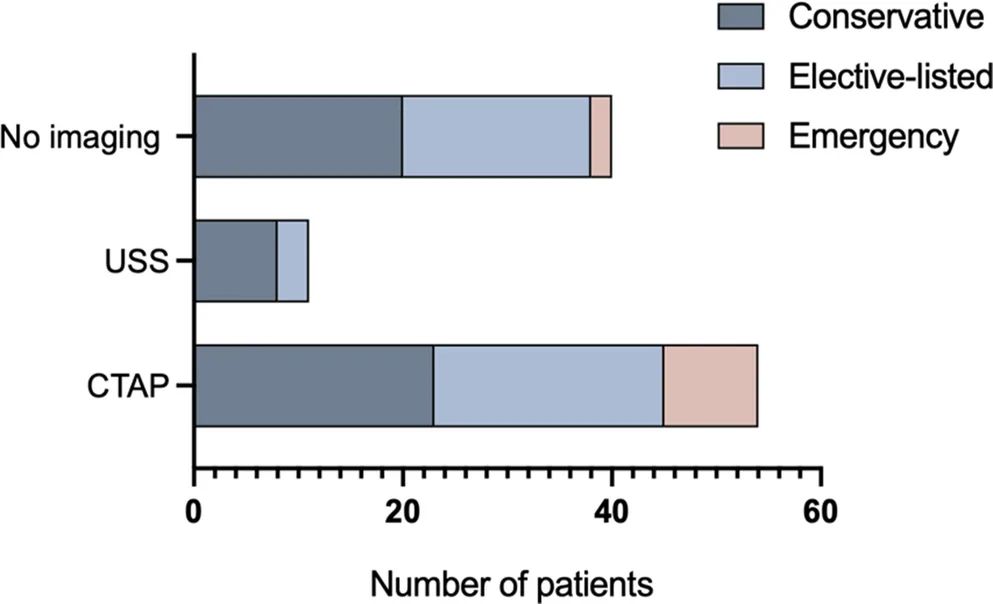
Distribution of management strategies by image modality

The most frequently used imaging modality was computed tomography scan of the abdomen and pelvis (CTAP) (*n* = 54, 51.4%) and 11 patients (10.5%) were assessed using ultrasound imaging (Fig. 2). Majority of patients who underwent emergency surgery had CTAP (*n* = 9, 81.8%) prior to operation. Furthermore, no diagnostic imaging was performed in 38.1% (*n* = 40) of patients. 

Of the 94 patients who did not undergo immediate surgery during presentation (watchful waiting and elective-listed), outcomes prior to definitive treatment are summarised in (Table 3). Patients managed with watchful waiting were more likely to undergo surgical crossover than those listed and awaiting elective repair (33.3% vs. 2.3%; *p* < 0.001; Table 3). Within the WW cohort, inguinal hernias (*n* = 22) had crossover rates of 9.1% to emergency surgery (2/22) and 40.9% to elective repair (9/22), giving a total crossover rate of 50.0%. Ventral hernias (*n* = 29) had crossover rates of 10.3% to emergency surgery (3/29) and 10.3% to elective repair (3/29), giving a total crossover rate of 20.7% (6/29).

**Table 3:** Outcomes for watchful waiting vs. elective-listed cohorts

	Watchful waiting (*n* = 51)	Elective-listed /awaiting surgery (*n* = 43)	p value*
Surgery crossover rate	33.3%	2.3%	< 0.0001
To emergency	5	1	
To elective (procedure completed)	5	0	
To elective (procedure pending)	7	n/a	
Hernia-related re-presentation			0.0172
< 30 days	8	3	
> 30 days	10	3	

Both cohorts were non-operative at this time of assessment; “Elective-listed” refers to patients listed for elective but awaiting surgery. *P* value *Fisher’s exact test

Emergency crossover from WW was primarily driven by worsening pain (*n* = 3) and new-onset strangulation or incarceration (*n* = 2). WW to elective crossover was most commonly prompted by outpatient clinic review (*n* = 8), pain (*n* = 2), or following outpatient ultrasound (*n* = 2). One of the elective-listed patients crossed over to emergency surgery for uncontrolled pain and presented with inguinal hernia. Additionally, 31.4% (*n* = 16) of WW patients were scheduled for outpatient follow-up clinic to reassess symptoms and discuss operative management, where 8 subsequently crossed over to be booked for elective surgery. A pre-existing, previously diagnosed hernia was present in 16 of 18 patients who experienced surgical crossed over.

**Fig. 3:**
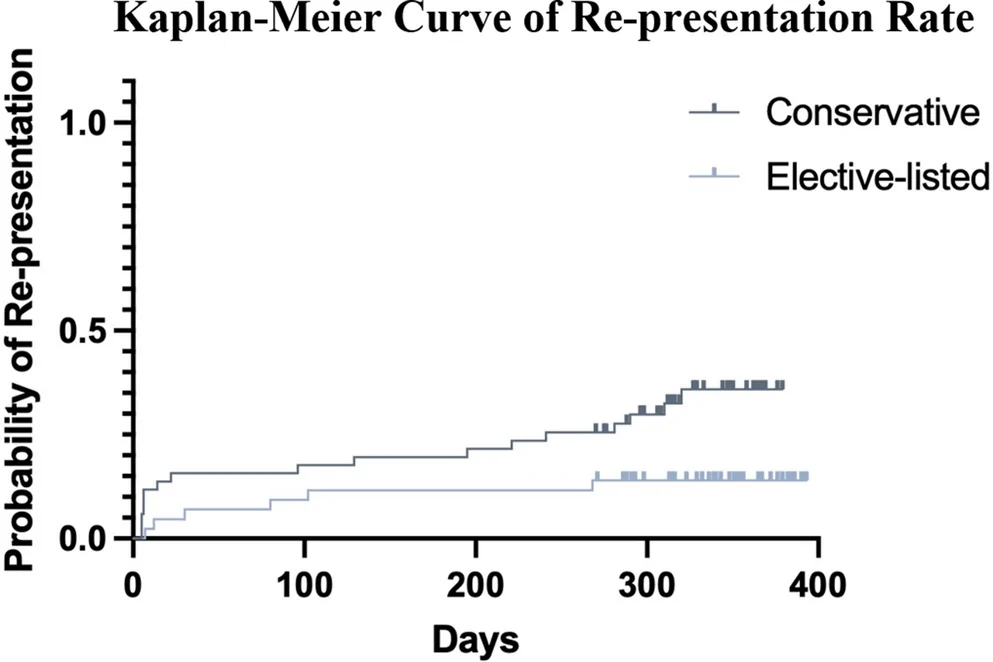
Kaplan-meier curve of re-presentation rate

Re-presentation to SAU was also notably higher in WW patients (35.3 vs. 14%, *p* = 0.0172, Table 2). In addition, pain requiring re-presentation to the SAU was also reported higher in 17.6% (*n* = 9) of WW patients compared with 14% (*n* = 6) of those awaiting elective repair. There was a 22.9% re-presentation rate (*n* = 24) over a follow-up period of 10.9 months. Kaplan–Meier analysis was used to evaluate time to re-presentation in the conservative WW and elective-listed groups, as shown in Fig. 3. Comparison of the curves using the log-rank test demonstrated a significant difference between groups (χ² = 4.709, *p* = 0.030), with a greater cumulative probability of re-presentation in the watchful waiting group over follow-up.

Among patients listed for elective repair who underwent surgery during the study period, the median waiting time to surgery was 43 days (IQR 17–122.5). Re-presentation rates were then analysed across observed waiting-time categories among all elective-listed patients, including patients who were still awaiting surgery by the end of the study period. Re-presentation occurred in 2/12 patients (16.7%) waiting ≤ 1 month, 2/10 patients (20.0%) waiting between 1 and 3 months, 1/8 patients (12.5%) waiting between 3 and 6 months, and 1/13 patients (7.7%) waiting > 6 months. Re-presentation rates did not differ significantly across waiting-time categories (Fisher’s exact test, *p* = 0.8772). 

By the end of study period, a total of 36 patients subsequently underwent elective surgery, while 17 patients underwent emergency surgery (including the crossed over patients). Post-operative outcomes were compared between two groups demonstrated in (Table 4). Median length of stay (LOS) is significantly longer for patients who underwent emergency surgery compared to elective for both ventral and inguinal hernias. The prolonged-stay patient presented with an incisional hernia. Median LOS was 0.5 days (IQR 0–2.25) with this case included. A sensitivity analysis excluding this patient yielded a median LOS of 0 days (IQR 0–2), indicating that the case did not substantially affect the interpretation of the LOS findings. Surgical complications were more frequent and more severe based on the Clavien-Dingo system for the emergency cohort for ventral hernias, consisting of one anastomotic leak who subsequently underwent a relook laparotomy with washout and side to side small bowel anastomosis.

**Table 4:** Outcomes following elective and emergency repair stratified by hernia type

	Ventral Hernia: Elective (Operated) (*n* = 14)	Ventral Hernia: Emergency (*n* = 9)	*p* value*	Inguinal Hernia: Elective (Operated) (*n* = 22)	Inguinal Hernia: Emergency (*n* = 8)	*p* value*
Length of staydays, median (IQR)	0.5 (0–2.25)	4 (2–13)	0.0079**	0 (0–0)	1.5(1–6)	0.0007**
Post-operative complication	4 (29%)	4 (44%)	0.6570	2 (9%)	2 (25%)	0.2835
Wound infection	0	0		1	0	
Seroma	2	0		1	0	
Intra-abdominal collection	0	1		0	0	
Anastomotic leak	0	1		0	0	
Large bowel obstruction	0	1		0	0	
Ileus	1	1		0	0	
Urinary retention	0	0		0	1	
Hernia recurrence	1	0		0	1	

*p* value *Fisher’s exact test, except **Non-parametric Mann-Whitney U test

Results also showed that open surgery was the predominant approach for emergency repair (Table 5). In contrast, a wider range of techniques were used electively, comprising of 63.9% open repair, 11.1% laparoscopic, and 22.2% robotic/robotic-assisted procedures. Two cases involved procedures outside standard hernia repair including laparoscopic loop ileostomy with stoma formation for spigelian hernia and ileostomy reversal for para-stomal hernia.

**Table 5:** Surgical approaches stratified by hernia type

	Ventral Hernia: Elective (Operated) (*n* = 14)	Ventral Hernia: Emergency (*n* = 9)	Inguinal Hernia: Elective (Operated) (*n* = 22)	Inguinal Hernia: Emergency (*n* = 8)
Open hernia repair	12	8	11	7
Laparoscopic procedure	0	0	4	1
Robotic/Robotic assisted	1	0	7	0
Reversal ileostomy	1	0	0	0
Laparoscopic loop ileostomy with stoma	0	1	0	0

## Discussion

The study population demonstrated an overall male predominance and was characterised by older age, with a median Charlson Comorbidity Index of 3 (min-max, 0–8), reflecting the possible baseline clinical complexity of patients presenting with acutely symptomatic hernias. In adjusted analysis, increasing age was a positive predictor of allocation to watchful waiting than elective surgery, suggesting that older patients may have been preferentially managed non-operatively due to perceived operative risk. Existing studies and the UK National Getting It Right First Time (GIRFT) programme also suggest that age, gender and co-morbidity are important factors during decision-making for identifying patients suitable for surgical repair, particularly whom may be associated with higher operative risk and those who may experience poorer outcomes [12–15].

A large proportion of patients in our cohort had a previously known hernia before their first acute presentation within the study period, including 16/18 of patients of those who crossed over to surgery. These findings suggest that untreated hernias may have an impact on the quality of life and daily functioning where surgical treatment could reduce subsequent healthcare utilization [8, 16]. However, the high prevalence of previously known hernia suggests that our cohort may reflect symptom progression among patients with established disease, representing a more symptomatic group in whom clinicians may defer operative repair pending appropriate medical optimisation [17]. GIRFT recommendations on inguinal and para-umbilical hernias recommend upfront waiting-list review to identify recurrent hernias and larger hernias that may not be suitable for the standard pathway, suggesting that these cases may require a more individualised approach because of greater baseline risk [14, 15]. Previous hernia history may therefore be a useful factor in risk stratification during decision-making in emergency surgical assessment.

At the same time, other parameters determining surgical outcomes such as frailty, ASA grade, and quality of life were difficult to acquire subjecting to measurement variability, as some of those parameters were only available for surgical patients and were not routinely recorded for patients managed with watchful waiting [18]. A recent clinician preference survey reported that patients with ASA grade 3 or 4 were more likely to undergo watchful waiting rather than surgical repair [10]. Hence routine documentation with accurate clinical coding may be able to strengthen triage process and provide a more complete adjustment for baseline operative risk and outcome prediction.

Acute hernia presentations are often complex, and patients initially managed non-operatively may later re-present and require surgery. Among those managed with watchful waiting, 9.8% later required emergency surgery and 23.5% were agreed for elective repair. Limited studies have examined this patient population. A 2017 study of 111 patients discharged from the emergency department with acutely symptomatic hernias (without indications for immediate repair) reported a similar 8% crossover to emergency surgery [19]. Reasons included worsening pain, strangulation, and incarceration. A subsequent multi-centre study reported a 5% emergency surgery rate with similar contributing factors [17]. Together, these findings suggest that a subset of patients remain at risk of emergency presentation despite non-operative management. However, both studies were conducted in the United States, where insurance status was reported to influence re-presentation and emergency surgery risk, limiting direct comparison with the UK public healthcare setting [17, 19].

To improve interpretability, we have stratified comparison by hernia types, and a literature search was also conducted in PubMed data base for studies reporting surgery crossover rates in inguinal and ventral hernias (Table 6). In the current inguinal subgroup, reasons for WW management were based on clinicians’ decisions, including older age, uncontrolled co-morbidity (e.g. diabetes, obesity and smoking), patient choice, planned outpatient review, hernia characteristics and reducible hernia with resolving pain [17]. The 9.1% crossover to emergency surgery suggests that, in some patients, watchful waiting may delay rather than avoid surgery. However, prior watchful-waiting studies in inguinal hernias often involve asymptomatic or minimally symptomatic patients, which has proven cost-effectiveness and safety for the implementation for WW, despite many of whom eventually progressed to surgery after up to ten years of follow-up [20–25]. These findings are therefore more informative, rather than being directly comparable with the present study.

**Table 6:** Summary of studies comparing surgical cross-over rates in inguinal and ventral hernias

Author	Study design	Duration of Study	Type of hernia	Cross-over rate	Reasons for cross-over
Fitzgibbons et al., [20]	RCT	2-4.5 years	Inguinal hernia	WW to surgery: 23% Acute hernia strangulation: 0.3%	Pain Symptoms limiting activity
O’Dwyer et al., [38]	RCT	574 days	Inguinal hernia	WW to surgery: 23/160 (14.4%)	Pain Progressive hernia protrusion
Chung et al., [22]	RCT	7.5 years	Inguinal hernia	WW to surgery: 16% (Year 1); 54% (Year 2); 72% by 7.5 years WW to emergency: 2/160 (1.25%)	Pain Symptoms affecting QoL Risk of incarceration
Fitzgibbons et al., [21]	RCT	7 years	Inguinal hernia	WW to surgery: 68%	Pain Patient-reported symptom burden Incarceration
de Goede et al., [23]	RCT	6 years	Inguinal hernia	35.4% WW to elective; 2.3% WW to emergency	Pain Hernia complication Cosmetic/aesthetic reasons
Cirocchi et al., [25]	Meta-analysis	N/A	Inguinal hernia	WW to elective surgery: between 35.03% − 57.8%	N/A
Ceresoli et al., [29]	Retrospective cross-sectional cohort study	3 years	Inguinal hernia	Elective to emergency: *n* = 17 patients (1.4%)	N/A
Van den Dop et al., [24]	RCT	12 years	Inguinal hernia	WW to surgery: 64.2%; WW to emergency: 3.9%	N/A
Yeow et al., [39]	Meta-analysis of RCT	N/A	Inguinal hernia	WW to surgery: Median cumulative rate: 54.2%, 1-year cumulative rate: 28.7%	Pain Incarceration
Kokotovic et al., [40]	Retrospective single-centre cohort study	5 years	Ventral (incisional and umbilical/epigastric) hernia	WW to emergency: 4% for both incisional and umbilical/epigastric hernias	N/A
Bernardi et al., [41]	Prospective study	3 years	Ventral hernia	Non-operative to surgery: 15/40 (37.5%)	N/A
Wolf et al., [42]	State-transition microsimulation model	N/A	Ventral hernia	WW to surgery: 39%; WW to emergency:14%	N/A
Dhanani et al., [27]	Prospective study	5 years	Ventral hernia	WW to surgery: *n* = 20 (50%); WW to emergency: *n* = 3 (15%)	Patient became more suitable for surgery following comorbidity optimisation Recurrent hernia-related symptoms Incarceration Concomitant surgery
Martin et al., [35]	Prospective study	3 years	Ventral hernia	WW to surgery: *n* = 31 (36.9%); WW to emergency: *n* = 7 (8.3%)	N/A
Holihan et al., [26]	Prospective observational study	12.2 months	Ventral hernia	23.2% surgical crossover from WW, with 3% to emergency and 20.2% to elective	Obstruction Incarceration Pain Patient medically optimised for surgery
Verhelst et al., [34]	Retrospective study	5 years	Incisional hernia	33% surgical crossover from WW to surgery. 8(24%) crossed over to emergency repair at a median of 1 month, and 13(38%) crossed over to elective operational treatment.	Incarceration Pain Patient preference Patient medically optimised for surgery Aesthetic complaints
Dadashzadeh et al., [28]	Observational study	8 years	Incisional hernia	Incarceration occurred in 540/23,022 (2.3%) patients, with cumulative incidence at 1.24% at 1 year and 2.59% at 5 years; WW to elective crossover occurred in 21.9% within 3 months and 9.8% between 3 months and 5 years.	Incarceration

*RCT *Randomised Controlled Trial, *WW *Watchful Waiting

Within this cohort, the overall crossover rate for ventral hernias was 20.7%, similar to the 23.2% reported in a prior study with a comparable follow-up period [26]. Patients who crossed over from non-operative management were also reported to experience improved satisfaction and function, suggesting that operative repair may benefit selected ventral hernia patients [26]. However, the available ventral literature remains limited and is composed largely of mixed cohorts. Among ventral hernia patients with co-morbidities, the reported 5-year crossover rate to surgery is 50%, including a 15% rate of emergency surgery [27]. For incisional hernias specifically, the reported risk of an acute hernia event at one year was 1.24% while 9.8% of patients ultimately required elective repair at 5 years, factors such as older age, female sex, higher BMI, and active smoking have been identified as independent predictors of non-operative management failure due to incarceration [28]. These findings highlight the importance of identifying patients with elevated perioperative risk who may still benefit from elective repair, as well as determining the optimal timing of surgery in high-risk patients [27].

Re-presentation occurred in both the WW and elective-listed groups, most commonly due to worsening hernia symptoms, pain, or emergency features. It was more frequent among patients managed with watchful waiting, as patients on the elective waiting list may have had lower re-presentation rates because a definitive operative plan was already in place, potentially providing greater reassurance and a clearer pathway for care. Previous studies have reported Emergency Department (ED) re-attendance rates of 18–25% among patients with symptomatic hernias as positive predictors of emergency surgery [17, 19]. Given that emergency surgery can still occur while patients await elective hernia repair, we examined whether longer waiting time was associated with unplanned re-presentation. However, among elective-listed patients within our cohort, longer waiting time was not significantly associated with re-presentation (*p* = 0.8772). Similarly, Ceresoli et al. also reported no association between prolonged waiting for elective groin hernia repair and emergency surgery [29]. Nevertheless, re-presentation can be underestimated, as patients may have sought care through primary care, private clinics, or other hospitals not captured within our institutional records.

Length of stay was significantly longer after emergency surgery than elective repair for both inguinal and ventral cohorts, which mirrors with the national MASH study which reported a median of 2 days, IQR 1–5 for acute presentations [2, 8]. 51.4% of patients underwent CT scan during their presentation compared to a lower rate of 10.5% using ultrasound, this contrast in imaging utilisation demonstrates the possible unavailability of ultrasonography out of hours. Complications were more frequent in the emergency cohort, likely reflecting the less controlled operative conditions of emergency hernia repair, where bowel resection, infection and contamination may contribute to longer-term post-operative morbidity [30, 31]. By contrast, elective repair allows a broader range of techniques, including laparoscopic and robotic approaches, which have been reported to associate with reduced postoperative pain and relatively faster recovery [2]. However, laparoscopic repair is not widely chosen within our cohort, aligning with the MASH study where only 17% of patients with unilateral groin hernia were offered laparoscopic repair [8]. This result indicates possible lack of training experience and highlights re-evaluation of minimally invasive surgery approach for acute hernias repairs [6, 8].

A further finding was the limited use of outpatient follow-up clinic for WW patients: only 31.4% were offered clinic review to reassess symptoms and discuss operative options. This may represent a potential missed opportunity for shared decision-making, as existing studies suggest that selected older or higher-risk patients may still benefit from elective repair rather than being excluded based on age or frailty alone [32, 33]. Among those reviewed, 50% were subsequently referred for elective repair. In practice, these decisions may have reflected symptom progression, patient preference, recurrent pain and reassessment of operative suitability [34]. Structured surveillance may therefore support timely escalation, by helping to identify a potential safe window for risk-reduction and preoperative optimisation in high-risk patients [35]. Patient education may also play an important role in optimising outcomes [17]. Limited awareness of hernia complications may contribute to delayed presentation, increasing the risk of emergency admission [31, 36]. Improved counselling with clear communication and safety-netting, supported by written information and better analgesia education may help mitigate such issues [37].

The follow-up period in this study was approximately 10.9 months. Although this duration was sufficient to assess early crossover and re-presentation, it may not fully reflect the longer-term course of watchful waiting. Over longer follow-up, patients who remain non-operative may not necessarily be clinically stable or free from ongoing hernia-related burden, as some patients within the WW cohort, particularly older patients, could later potentially become less suitable for surgery due to increasing frailty or comorbidity. In this context, elective repair, when clinically suitable, may represent a reasonable option in selected patients as part of a balanced management strategy to help reduce future hernia-related burden. 

### Study limitations

Our findings should be interpreted considering several limitations. As a single-centre observational study, it demonstrates possible associations rather than causal relationships, with smaller cohort and limited timeframe, affecting overall generalisability. Management allocation was clinician-directed rather than randomised, introducing the possibility of potential confounding. This study also included patients with a previously known hernia, some of whom may have received prior surgical or non-surgical management elsewhere, which may have introduced selection bias and potentially inflated the observed crossover rates.

Inclusion of both inguinal and ventral hernias may have masked differences between individual hernia subtypes by introducing a heterogenous mix, and more features of hernia complexity, such as defect size, inguinoscrotal extension, loss of domain, or multiple defects, were not consistently documented, limiting further adjustment for anatomical complexity. We have stratified by hernia type and performed a subgroup analysis to explore variation while maintaining the broader applicability of the cohort. However, these results remain hypothesis generating, and outcomes is likely to differ across specific hernia subgroups, warranting further studies with detailed documentation and longer follow-up. Acute hernia presentations ranged from pain alone to emergency complications, and variation in pain thresholds, symptom tolerance, and thresholds for seeking urgent care may have further contributed to heterogeneity within the cohort. Nevertheless, this study may contribute to existing literature by capturing consecutive acute presentations and reflecting real-world decision-making under routine service constraints, generating evidence that might be difficult to obtain within RCT settings [40].

## Conclusion

This single-centre retrospective study describes the management pathways and early outcomes of patients presenting with acutely symptomatic hernias. Management decisions varied between watchful waiting, elective listing, and emergency surgery, with crossover and re-presentation observed during follow-up. Emergency repair was associated with longer length of stay and greater postoperative morbidity compared to elective surgery, particularly among patients with ventral hernias. Among patients managed with watchful waiting, limited outpatient follow-up and subsequent crossover to surgery suggest that more structured surveillance and reassessment may be beneficial in selected patients, supporting shared decision-making regarding the appropriateness of elective repair. Further multicentre studies with longer follow-up are needed to validate these findings, develop risk-stratification approaches, and identify patients at higher risk of future emergency surgery.

## Data Availability

All data relevant to this study are included in the article. Anonymised data may be available on reasonable request from the authors subject to institutional approval.
